# Pyridinedicarboxylate-Tb(III) Complex-Based Luminescent Probes for ATP Monitoring

**DOI:** 10.1155/2021/7030158

**Published:** 2021-08-10

**Authors:** Dien Nguyen Thi, Nhung Nguyen Thi, Anh-Tuan Vu, Thuong Quang Tran, Tue Nguyen Ngoc, Dien Luong Xuan, Thao Ta Thi, Truong Nguyen Xuan

**Affiliations:** ^1^School of Chemical Engineering, Hanoi University of Science and Technology, 01 Dai Co Viet, Hai Ba Trung, Hanoi 100000, Vietnam; ^2^Faculty of Chemistry, VNU University of Science, Vietnam National University, 19 Le Thanh Tong, Hoan Kiem, Hanoi 100000, Vietnam

## Abstract

The pyridinedicarboxylate-Tb(III) complexes, TbPDC and Tb(PDC)_3_, as luminescent probes for ATP monitoring have been conveniently prepared and characterized by FT-IR, ^1^H-NMR, ESI-MS, UV-Vis, excitation, and emission spectroscopy. Interestingly, these two Tb(III) complexes were quenched by ATP by a similar mechanism via *π*-*π* stacking interaction between the chelating ligand and adenine moiety. The ability of luminescent probes applied for the determination of ATP in aqueous solution has been investigated. The dynamic ranges for the quantification of ATP are within 10−90 *μ*M and 10−100 *μ*M with detection limits of 7.62 and 11.20 *μ*M for TbPDC and Tb(PDC)_3_, respectively. The results demonstrated that these luminescent probes would be a potential candidate assay for ATP monitoring in hygiene assessment.

## 1. Introduction

Adenosine-5′-triphosphate (ATP) is known as an energy-carrying molecule within living cells. ATP concentration has also been widely used for the assessment of the hygienic state or cleanliness of contact surfaces in healthcare settings, food quality control, and environmental analyses [1–3]. For hygiene monitoring, the most commonly used method for the determination of ATP is the bioluminescence method based on the assumption that the amount of the microbial biomass is directly proportional to the amount of ATP in the sample. ATP-based bioluminescence assay has become a valuable technique for healthcare-associated infections (HAIs) and for food safety management procedures, particularly as part of the general hazard analysis and critical control point (HACCP) measurements [2, 3]. In addition to bioluminescence methods, a variety of lanthanide-based luminescent probes have been developed for the real-time detection and quantification of ATP [4–7]. Lanthanide complexes, especially Tb^3+^- and Eu^3+^-complexes, have been widely used as luminescent probes due to their unique optical properties, such as large Stokes shifts, long luminescence lifetimes (up to ms), and narrow emission bands [7–11]. They are synthetically designed in several ways to display photophysical changes in the presence of an analyte. Probably, one of the most common types is the lanthanide complex with an antenna linked with the chelating ligand which allows an analyte to be bound [9]. In another type, the lanthanide complex is coordinatively unsaturated. The remaining coordination sites are occupied by weakly bound solvent molecules. As a result, the solvent molecules are displaced in the presence of competing analytes [8, 12]. A great number of such luminescent lanthanide complexes have been designed using chromophores containing pyridine [13–16], carboxylate [17, 18], *ß*-diketone [19, 20] group, fluorescent dye or its derivatives, etc. There are various mechanisms which explain for the changes of luminescence of those lanthanide-based probes including binding effect, electrostatic interaction, intramolecular charge transfer (ICT), photoinduced electron transfer (PET), and luminescence resonance energy transfer (LRET) [7, 10]. ATP consists of an adenine, a ribose, and a triphosphate group, of which adenine, triphosphate, or both of them are capable of involving interaction mechanisms with luminescent lanthanide-based probes. For example, Pierre and coworkers [21] reported that the adenine moiety can participate in favorable *π*-*π* stacking interaction with the phenanthridine moiety, via PET, resulting in a decrease of luminescent intensity of the Eu^3+^-probe. Schäferling et al. [22] suggested that only the phosphate part of ATP participates in binding to the Eu^3+^-probe. The authors [5, 12] indicated that both adenine and phosphate parts of ATP covalently bind to the synthesized lanthanide probes owning the self-assembled spherical [5] or helical dinuclear structure [12].

In this contribution, we have prepared the pyridinedicarboxylate-Tb(III) complexes and investigated their interactions with ATP. The structure and photophysical properties of the complexes were reported. The complex's luminescent responses in the presence of ATP have been studied to evaluate its possibility in using as a probe for ATP monitoring.

## 2. Experimental

### 2.1. Materials

Terbium(III) carbonate hydrate (99.99%, Alfa Aesar), pyridine-2,6-dicarboxylic acid (99%, Sigma-Aldrich), adenosine-5′-triphosphate disodium salt hydrate (≥99%, Sigma-Aldrich), adenine (99%, Alfa Aesar), adenosine (≥99%, Sigma-Aldrich), tris(hydroxymethyl)aminomethane (≥99.8%), Na_2_CO_3_ (powder, ≥99.5%), Na_2_SO_4_ (powder, ≥99.0%), Na_3_PO_4_.12H_2_O (98.0–102.0%) were supplied by Merck, and other chemicals (analytically pure) were used without further purification.

### 2.2. Preparation of Pyridinedicarboxylate-Tb(III) Complexes

**Scheme 1:**
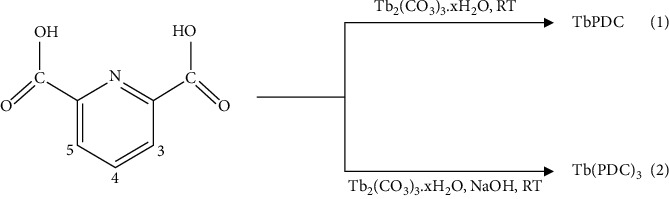
Synthetic procedures of the Tb(III) complexes: (pyridine-2,6-dicarboxylato)terbium (1^+^) (TbPDC) and tris(pyridine-2,6-dicarboxylato)terbium (3^−^) (Tb(PDC)_3_).

The pyridinedicarboxylate-Tb(III) complexes were prepared using the previously reported method with slight modifications (Scheme 1) [11]. 0.351 g (2.1 mmol) of pyridine-2,6-dicarboxylic acid (PDCA) was dissolved in 20 mL ethanol to prepare solution A. 0.176 g (0.35 mmol) of Tb_2_(CO_3_)_3_ (hydrated terbium carbonate) was dissolved in 20 mL ethanol to obtain solution B. The precipitate (compound 1) was obtained by mixing solutions A and B. Then, the precipitate was filtered, washed with ethanol, and dried at 60°C overnight. Yield was 0.179 g. Compound 2 was prepared in the same route except that the pH value of solution A was adjusted to 7 by adding sodium hydroxide (0.1 M aqueous solution) before mixing with solution B. Yield was 0.168 g.

### 2.3. Methods

Fourier-transform infrared (FT-IR) spectra were obtained using an IRAffinity-1S FT-IR spectrometer (Shimadzu, Japan). Proton nuclear magnetic resonance (^1^H-NMR) spectra were recorded in DMSO-d_6_ at room temperature using a Bruker AVANCE Neo 600 MHz spectrometer. Mass spectra were recorded using an LC-MSD Trap SL ion trap mass spectrometer with electrospray ionization (Agilent, USA). Absorption spectra were obtained using an ultraviolet-visible (UV-Vis) Agilent 8453 spectrophotometer.

All luminescence measurements were carried out on an F-4700 spectrofluorimeter (Hitachi, Japan). Luminescence quenching data were analyzed using the Stern–Volmer relation [23]:

(1) $$\begin{array}{c} \frac{F_{0}}{F}-1=K_{\text{SV}}\left[Q\right], \end{array}$$

where *F*_0_ is the initial luminescence intensity in the absence of ATP, *F* is the measured luminescence intensity, *K*_SV_ is the Stern–Volmer constant, and [*Q*] is the ATP concentration.

## 3. Results and Discussion

### 3.1. Characterization and Properties of the Pyridinedicarboxylate-Tb(III) Complexes

#### 3.1.1. FT-IR Spectroscopy

The main infrared absorption bands of the complexes and the assignments are listed in Table 1 (see Figure S1 for infrared spectra). All the bands involving carboxyl (–COOH) disappear [11, 15], but the asymmetric and symmetric stretching vibration frequencies (*υ*_as_ and *υ*_s_) of the carboxylate group (–COO^–^) are observed. It is concluded that all the ligands coordinated the terbium ion through their carboxyl. The bands with frequencies 1373 cm^−1^ (1) and 1369 cm^−1^ (2) are assigned to the stretching vibration of complexes' pyridine ring. The peaks at 428 cm^−1^ (1) and 420 cm^−1^ (2) are attributed to the stretching vibrations of N ⟶ Tb bonds [13]. It indicates that pyridine ring's nitrogen participates in coordination. The medium-intensity bands at 1076 cm^−1^ (1) and 1072 cm^−1^ (2) are assigned to the bending vibrations *δ*(=C−CH) [14].

#### 3.1.2. Mass Spectroscopy

Figure S2(a) shows the signals of the ligand (*m*/*z* = 166.7) and the ligand with terbium ions (*m*/*z* = 342.9), whereas the signals in Figure S2(b) indicate various forms of the complex (*m*/*z* = 342.9 and 671.2) (Table 2). This suggests that the complex was formed with a 1 : 1 stoichiometry (TbPDC, 1) in the first synthesis procedure, whereas the complex with 1 : 3 stoichiometry (Tb(PDC)_3_, 2) was formed in the second one. Different conformations of complexes are probably related to the efficient proton abstraction from ligands by solvent molecules. Alcohol has much weaker affinity than the water molecule for the proton.

#### 3.1.3. ^1^H-NMR Spectra Analysis

The ^1^H-NMR spectrum in Figure S3(a) shows one multiplet (br, 1H) around 8.440 − 8.236 ppm which was assigned to H4 of the pyridine ring. A broad peak at 4.452 ppm (s, 2H) in Figure S3(b) was assigned to H3 and H5 of the pyridine ring, which indicates that the Tb−O and Tb ⟶ N bonds are formed in the complex [15].

#### 3.1.4. Excitation and Emission Spectroscopy

**Figure 1:**
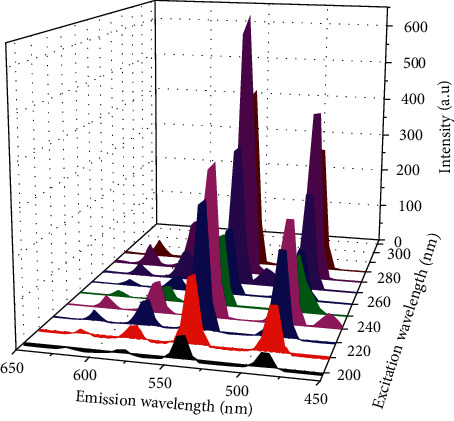
Excitation and emission spectra of 1 (24 mg/L) in Tris-HCl buffer (pH 7.4).

The excitation and emission spectra of the pyridinedicarboxylate-Tb(III) complexes in Tris-HCl buffer (pH 7.4) are shown in Figure 1 and Figure S4 with the intense transitions listed in Table 3. In the excitation spectra, the peaks at 225 and 270 nm are assigned to *π* ⟶ *π*^*∗*^ transitions of the ligands. The emission spectra exhibit four characteristic wavelengths of Tb^3+^ ions at 490, 545, 585, and 622 nm designated to ^5^D_4_ ⟶ ^7^F_6_, ^7^F_5_, ^7^F_4_, and ^7^F_3_, respectively [9, 10].

#### 3.1.5. UV-Vis Absorption Spectroscopy

**Figure 2:**
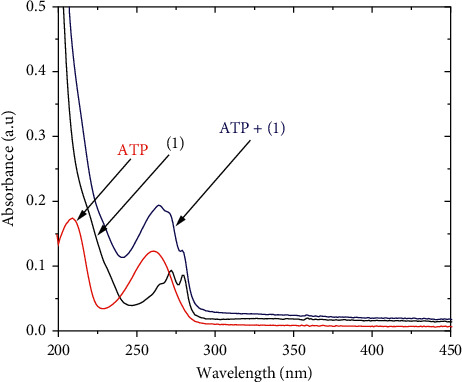
UV-Vis absorption spectra of 1 (24 mg/L) and ATP (10^−4^ M) in Tris-HCl buffer solution (pH 7.4).

The UV-Vis absorption spectra of the pyridinedicarboxylate-Tb(III) complexes and ATP in aqueous solution are shown in Figure 2 and Figure S5. All compounds exhibit broad, intense absorption bands between 220 and 300 nm. The peaks at the wavelength of 272 and 280 nm correspond to *π*-*π*^*∗*^ transitions of the ligand of the complexes that coincide with their observed excitation spectra. Furthermore, absorption spectra of the complexes recorded in the absence and in the presence of ATP reveal that there is no association between the complex and ATP in the ground state because the spectrum of the mixture is likely the additive spectrum of separate components.

### 3.2. Pyridinedicarboxylate-Tb(III) Complexes as Luminescent Probes for ATP Monitoring

#### 3.2.1. The Interaction of the Pyridinedicarboxylate-Tb(III) Complexes with ATP

**Figure 3:**
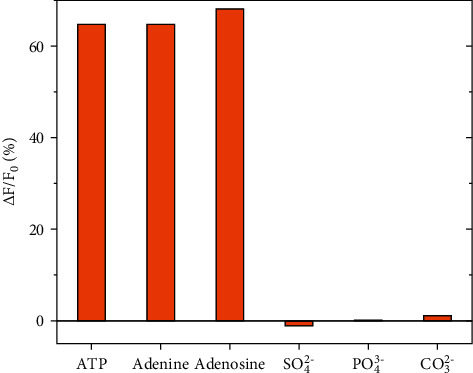
Influence of the presence of anion or neutral molecules (10^−4^ M) on the luminescence of 1 (24 mg/L; Tris-HCl buffer solution (pH 7.4)).

TbDPC (1) is coordinately unsaturated within the DPC ligand in which the uncoordinated sites are taken up by H_2_O molecules. It may be expected that this intentionally prepared complex (1) acts as a probe for anions which would replace the coordinating H_2_O molecules [8]. However, it is not experimentally observed in case of 1. The presence of anions such as PO_4_^3−^, CO_3_^2−^, or SO_4_^2−^ almost does not cause any change of the luminescent intensity of 1 (Figure 3). It is also worth to note that its luminescence might not be affected by the electrostatic interaction between cationic Tb^3+^ and these anions. On the contrary, it is interesting to find out that the quenching reaction takes place between 1 and ATP, a phosphate-containing anion, resulting in decreased complex's luminescence. This gives a hint that the *π*-*π* stacking rather than electrostatic attraction plays a role in their interaction. Indeed, the quenching of 1 (Figure 3) by adenine and adenosine, which are adenine-based structural molecules, has provided a proof for the above suggestion. In contrast to 1, Tb(DPC)_3_ (2) is fully coordinated within three DPC ligands, i.e., one nitrogen of the pyridine ring and two carboxyls in each. This type of complex also displays the same behaviors as 1 to the investigated substances including ATP, adenine, adenosine, and several anions (Figure S6). Thus, it leads to the conclusion that the quenching mechanism of the pyridinedicarboxylate-Tb(III) complexes by ATP involves the *π*-*π* interaction between the pyridine moiety and adenine moiety.

#### 3.2.2. ATP Monitoring

**Figure 4:**
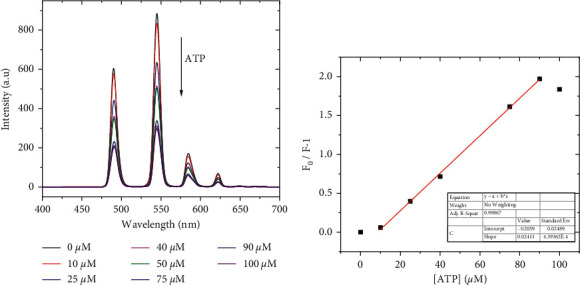
(a) Fluorescence spectra of 1 at various concentrations of ATP in Tris-HCl buffer solution (pH 7.4). (b) Stern–Volmer plot corresponding to the obtained data in (a).

The Tb(III) complexes, 1 and 2, were used as luminescent probes for ATP monitoring. The luminescence changes of probes following the continuous addition of ATP are seen in Figure 4(a) and Figure S7(a). It shows that the luminescent intensity of 1 or 2 decreased with an increase of ATP concentration. When the ATP concentration increased to 500 *μ*M, the luminescence was completely quenched, and a good linear relationship between the luminescent intensity and the concentration of ATP was observed, as shown in Figure 4(b) and Figure S7(b) for 1 and 2, respectively. The obtained data monitored at 545 nm were analyzed according to the Stern–Volmer relation (equation (1)), and other calculated analytical parameters are summarized together in Table 4, in which the limit of detection (LOD) was calculated as follows [24]:

(2) $$\begin{array}{c} \text{LOD}=\frac{3.3\sigma }{\text{slope}}, \end{array}$$

where *σ* and slope resulted from the linear fit according to equation (1).

## 4. Conclusion

The pyridinedicarboxylate-Tb(III) complexes (1 and 2) were prepared under mild conditions and characterized by various techniques. 1 consists of one pyridine-2,6-dicarboxylate unit, whereas 2 is fully coordinated by the three ligands. Although the Tb(III) complexes possess different configurations, their photophysical properties are relatively similar. They display characteristic emission wavelengths of Tb^3+^ ion following the excitation. In addition, 1 and 2 can be used as luminescent probes for ATP monitoring. The response characteristic reveals that the *π*-*π* stacking interaction between the adenine moiety of ATP and chelating ligand leads to the quenching effect of the complex. The linear ranges for the determination of ATP are within 10−90 *μ*M and 10−100 *μ*M, and the detection limits are 7.62 and 11.20 *μ*M for 1 and 2, respectively. Although improving sensitivity and selectivity remains a challenge, this work contributes a potential assay based on pyridinedicarboxylate-Tb(III) probes which can be applied to monitor ATP for hygienic assessment.

## Figures and Tables

**Table 1:** IR bands (cm^−1^) of the pyridinedicarboxylate-Tb(III) complexes.

Complex	Py	*δ*	*υ*_as_	*υ*_s_	*υ*
	(C=N)	(=C−CH)	(COO^−^)	(COO^−^)	(N ⟶ Tb)
TbDPC	1373	1076	1570	1435	428
Tb(DPC)_3_	1369	1072	1587	1437	420

**Table 2:** Complexes confirmed by the electrospray ionization-mass spectrometry (ESI-MS) method.

Fragmentation pattern (*m*/*z*)	Assignment
166.7	[PDC + 2H]^+^
342.9	[Tb(PDC) + H_2_O]^+^
671.2	[Tb(PDC)_3_ + H_2_O]^+^

**Table 3:** Luminescence properties of the pyridinedicarboxylate-Tb(III) complexes (24 mg/L) in Tris-HCl buffer (pH 7.4).

Complex	*λ*_ex_ (nm)	Assignment	*λ*_em_ (nm)	Assignment
TbDPC	225	*π* ⟶ *π*^*∗*^	490	^5^D_4_ ⟶ ^7^F_6_
	270	*π* ⟶ *π*	545	^5^D_4_ ⟶ ^7^F_5_
			585	^5^D_4_ ⟶ ^7^F_4_
			622	^5^D_4_ ⟶ ^7^F_3_

Tb(DPC)_3_	225	*π* ⟶ *π*^*∗*^	490	^5^D_4_ ⟶ ^7^F_6_
	270	*π* ⟶ *π*	545	^5^D_4_ ⟶ ^7^F_5_
			585	^5^D_4_ ⟶ ^7^F_4_
			622	^5^D_4_ ⟶ ^7^F_3_

Emission spectra at *λ*_ex_ = 270 nm and *λ*_ob_ = 545 nm; emission and excitation slit widths: 5 nm; PMT voltage: 400 V.

**Table 4:** Analytical parameters for ATP monitoring by pyridinedicarboxylate-Tb(III) probes (24 mg/L) in Tris-HCl buffer (pH 7.4).

Complex	*K*_SV_ (*μ*M^−1^)	Linearity (*μ*M)	LOD (*μ*M)
TbDPC	0.0241	10–90	7.62
Tb(DPC)_3_	0.0278	10–100	11.20

## Data Availability

The data supporting the findings of this study are available within the article and its supplementary materials.

## Supplementary Materials

Supplementary Materials Supplementary Figures S1, S2, and S3 provide the FT-IR, ESI-MS, and ^1^H-NMR spectra of complexes 1 and 2, respectively. The excitation and emission spectra of 2 and the interaction between 2 and ATP are shown in Figures S4 and S5 (UV-Vis spectra). The influence of strange substances and ATP on the luminescent intensity of 2 and the corresponding Stern–Volmer plot are described in Figures S5, S6, and S7, respectively.
