# Supplementary material for: Pyridinedicarboxylate-Tb(III) Complex-Based Luminescent Probes for ATP Monitoring
Source: J Anal Methods Chem. 2021 Aug 10;2021:7030158. doi: 10.1155/2021/7030158 (PMC8370815; doi:10.1155/2021/7030158)
Supplement: Supplementary Materials — Supplementary Figures S1, S2, and S3 provide the FT-IR, ESI-MS, and 1H-NMR spectra of complexes 1 and 2, respectively. The excitation and emission spectra of 2 and the interaction between 2 and ATP are shown in Figures S4 and S5 (UV-Vis spectra). The influence of strange substances and ATP on the luminescent intensity of 2 and the corresponding Stern–Volmer plot are described in Figures S5, S6, and S7, respectively. [file 7030158.f1.doc]

**Supplementary Materials**

**Pyridinedicarboxylate-Tb(III) Complexes based Fluorescent Probes for ATP Monitoring**

**Dien Nguyen-Thi^1^, Nhung Nguyen-Thi^1^, Tuan Vu-Anh^1^, Quang Tran-Thuong^1^, Tue Nguyen-Ngoc^1^, Dien Luong-Xuan^1^, Thao Ta-Thi^2^, Truong Nguyen-Xuan^1^**

*^1^School of Chemical Engineering, Hanoi University of Science and Technology, 01 Dai Co Viet, Hai Ba Trung, Hanoi 100000, Vietnam.*

*^2^Faculty of Chemistry, VNU University of Science, Vietnam National University, 19 Le Thanh Tong, Hoan Kiem, Hanoi 100000, Viet Nam*

*^*^Email: truong.nguyenxuan@hust.edu.vn*

The Supplementary Materials provides supporting figures described in the paper. For characterization of the complexes **1** and **2**: FT-IR (Fig. S1), ESI mass (Fig. S2), ^1^H-NMR spectra (Fig. S3), for photophysical properties of **2:** excitation and emission spectra (Fig. S4), and for the interaction between **2** and ATP: UV-Vis spectra (Fig. S5), the influence of strange substances (Fig. S6) and ATP (Fig. S7.a), and the Stern-Volmer plot (Fig. S7.b).


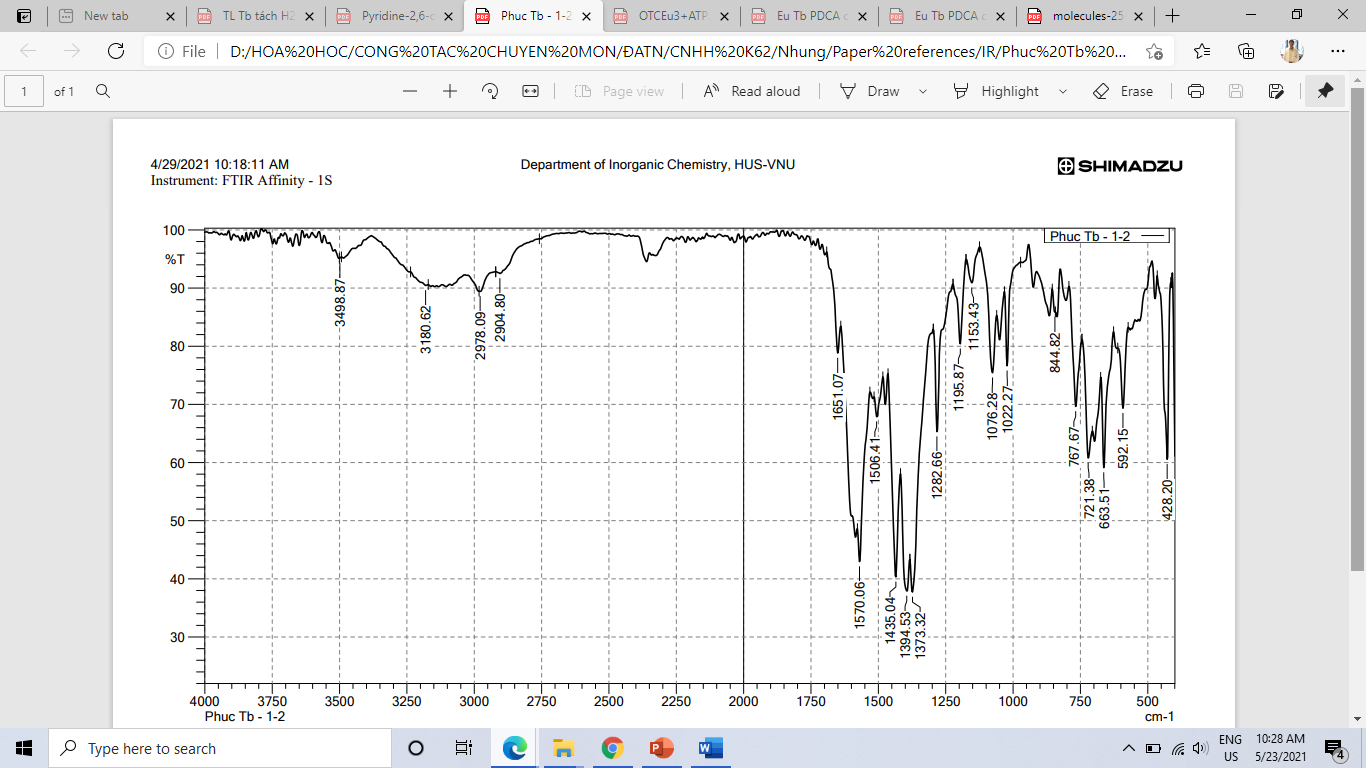


**(A)**


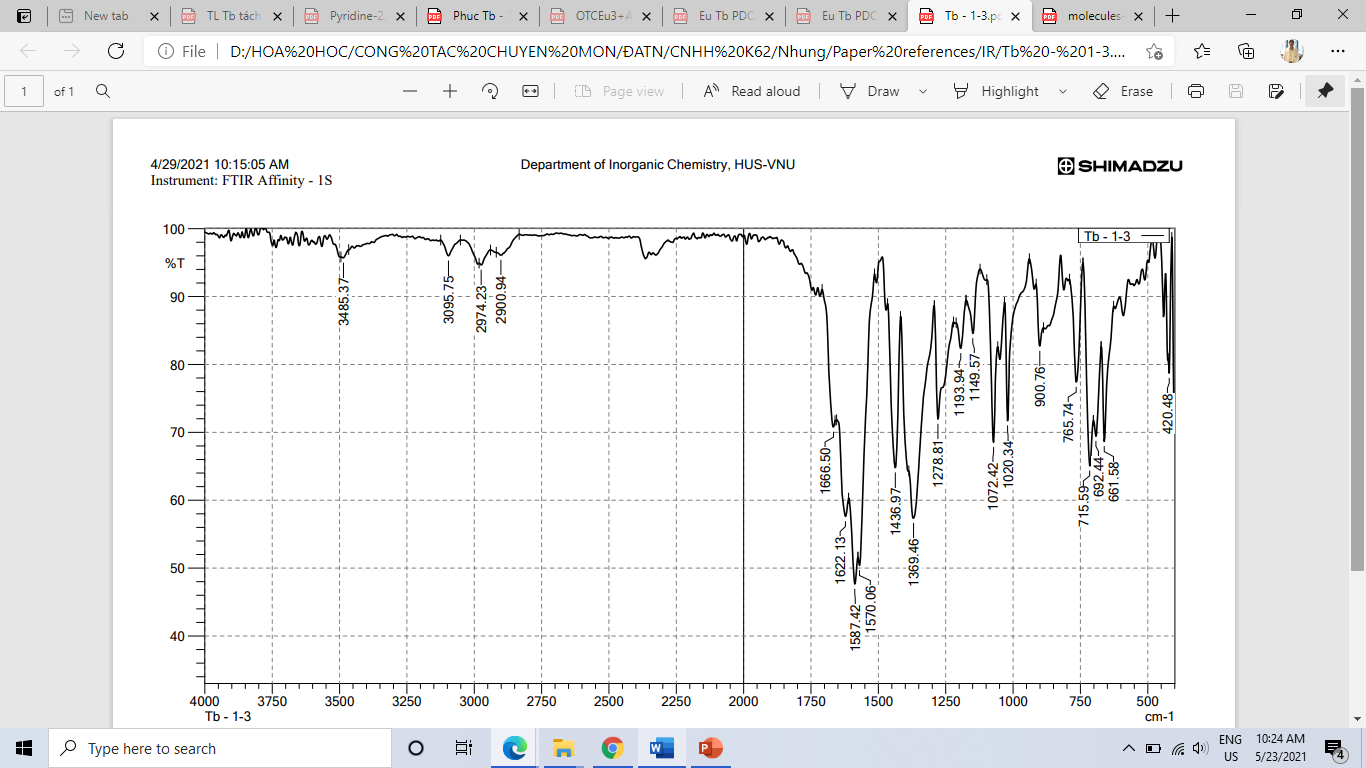


**(B)**

Fig. S1. Infrared spectra of complexes: **1** (A) and **2** (B).


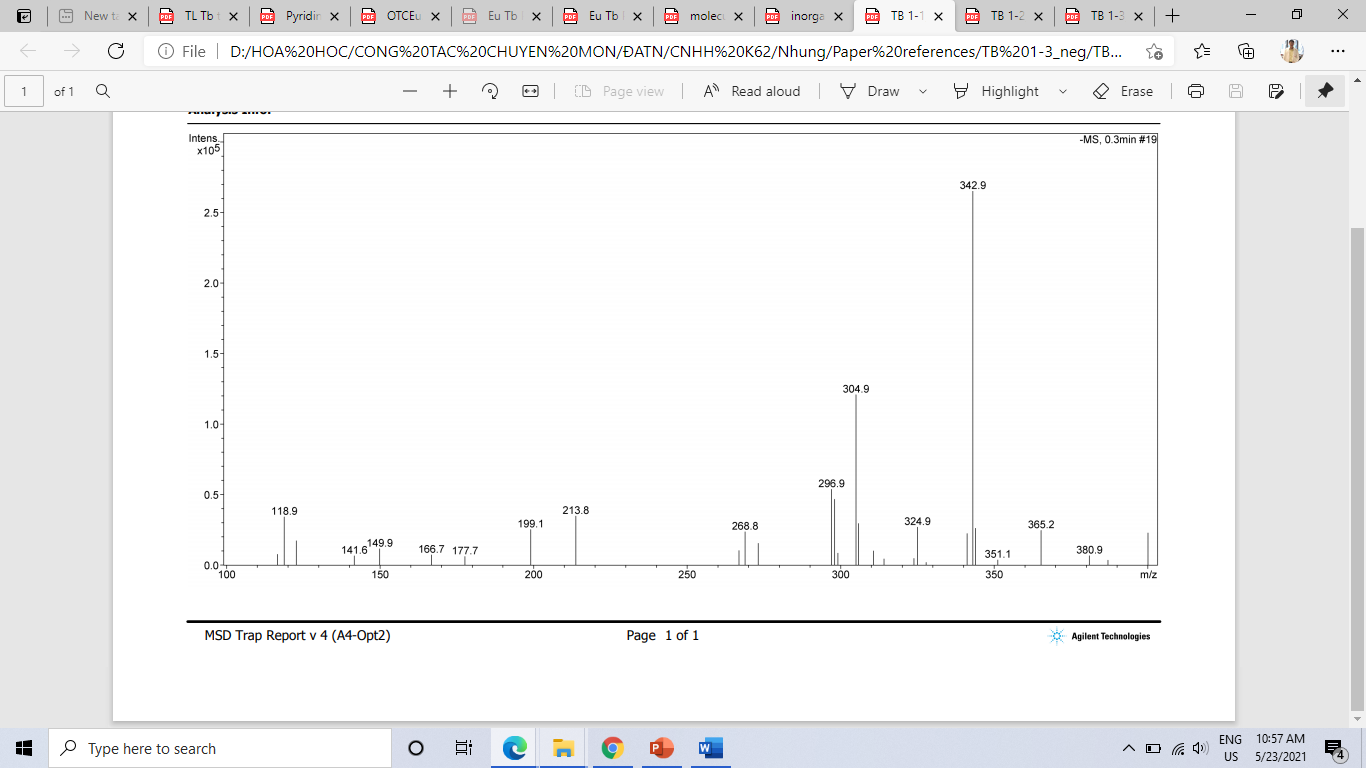


**(A)**


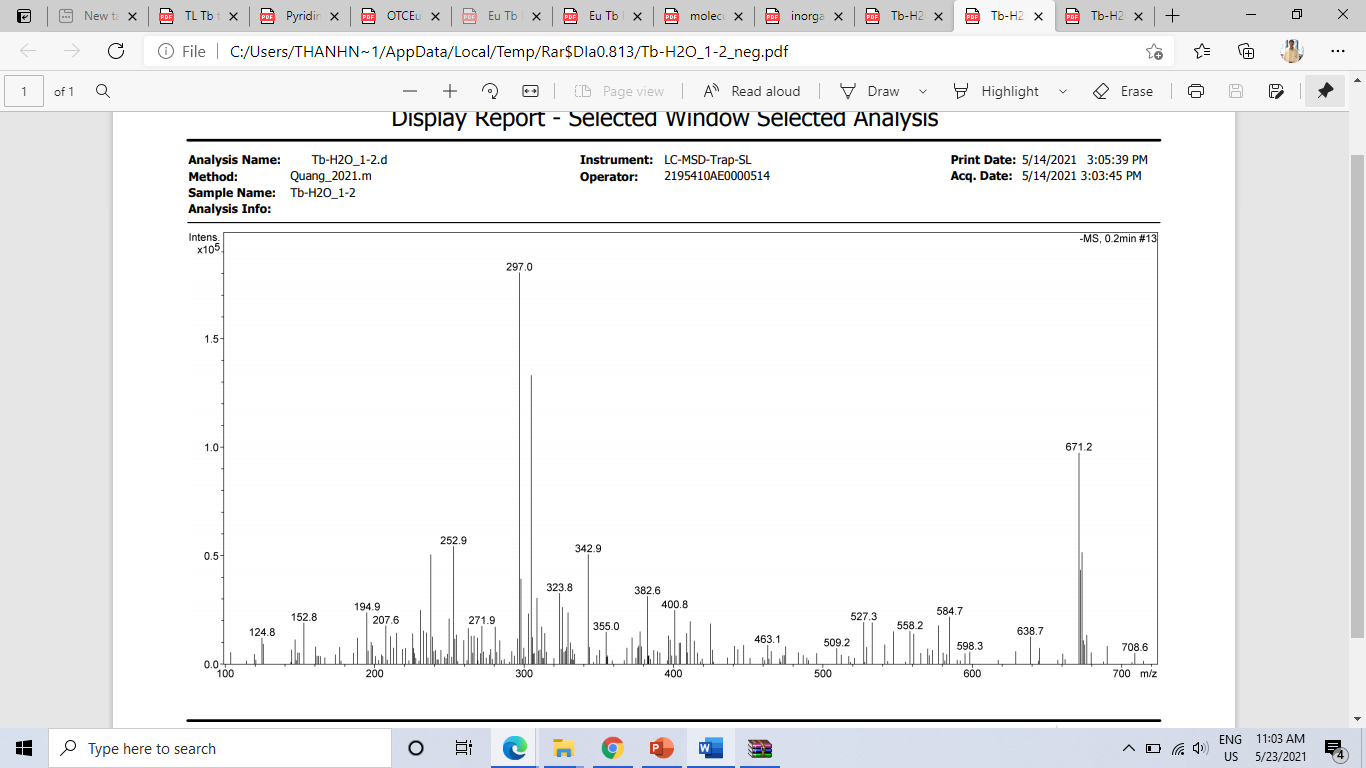


**(B)**

Fig. S2. The ESI mass spectra of investigated complexes: **1** (A) and **2** (B).

| **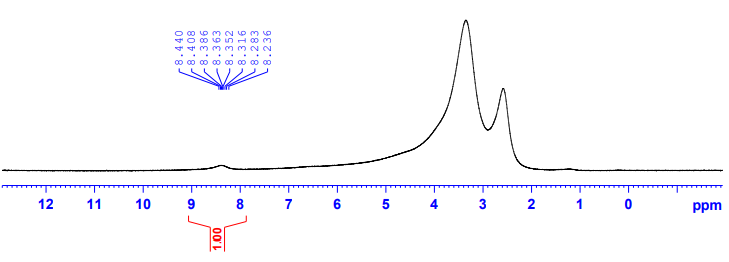**  **(A)** | **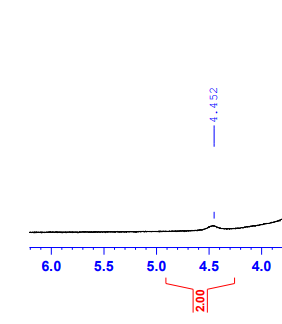**  **(B)** |
| --- | --- |
| Fig. S3. ^1^H-NMR spectra of **1** (A) and **2** (B) in DMSO-D_6_. | |

Fig. S4. Excitation and emission spectra of **2** (12 mg/L) in Tris-HCl buffer pH 7.4

Fig. S5. UV-Vis absorption spectra of **2** (24 mg/L) and ATP in Tris-HCl buffer solution pH 7.4.

Fig. S6. Influence of the presence of anion or neutral molecules to the luminescence of **2** (24 mg/L; Tris-HCl buffer solution pH 7.4).

Fig. S7.a. Luminescence spectra of **2** (24 mg/L) at various concentrations of ATP in Tris-HCl buffer solution pH 7.4.

Fig. S7.b. Stern-Volmer plot corresponding to the obtained data in Fig. S7.a.
